# Feasibility of the Dutch ICF Activity Inventory: a pilot study

**DOI:** 10.1186/1472-6963-10-318

**Published:** 2010-11-26

**Authors:** Janna E Bruijning, Ruth MA van Nispen, Ger HMB van Rens

**Affiliations:** 1 Department of Ophthalmology, VU University Medical Center, PO Box 7057, 1007 MB Amsterdam, The Netherlands; 2 EMGO Institute for Health and Care Research (EMGO+), VU University Medical Center Amsterdam, Van der Boechorststraat 7, 1081 BT Amsterdam, The Netherlands; 3 Department of Ophthalmology, Elkerliek Hospital, Wesselmanlaan 25, 5707 HA Helmond, The Netherlands

## Abstract

**Background:**

Demographic ageing will lead to increasing pressure on visual rehabilitation services, which need to be efficiently organised in the near future. The Dutch ICF Activity Inventory (D-AI) was developed to assess the rehabilitation needs of visually impaired persons. This pilot study tests the feasibility of the D-AI using a computer-assisted telephone interview.

**Methods:**

In addition to the regular intake, the first version of the D-AI was assessed in 20 patients. Subsequently, patients and intake assessors were asked to fill in an evaluation form. Based on these evaluations, a new version of the D-AI was developed.

**Results:**

Mean administration time of the D-AI was 88.8 (± 41.0) minutes. Overall, patients and assessors were positive about the D-AI assessment. However, professionals and 60% of the patients found the administration time to be too long. All included items were considered relevant and only minor adjustments were recommended.

**Conclusion:**

The systematic character of the revised D-AI will prevent topics from being overlooked and indicate which needs have the highest priority from a patient-centred perspective. Moreover, ongoing assessment of the D-AI will enhance evaluation of the rehabilitation process. To decrease administration time, in the revised D-AI only the top priority goals will be fully assessed. Using the D-AI, a rehabilitation plan based on individual needs can be developed for each patient. Moreover, it enables better evaluation of the effects of rehabilitation. A larger validation study is planned.

## Background

As a result of demographic ageing, the number of people with irreversible visual impairments is expected to rise in the coming decades [1,2]. This will lead to increasing pressure on visual rehabilitation services [3], which need to be efficiently organised in the near future. Therefore, the rehabilitation needs of visually impaired people have to be thoroughly investigated immediately after entering a multidisciplinary rehabilitation centre (MRC). This assessment must be performed based on the needs of the patient, not on the supply of the MRC. A patient-centred goal formulation structure in the rehabilitation process may increase patient motivation and help achieve better outcomes [4-6]. Therefore, it is essential that patients point out their own priorities. Many studies have focused on specific problems from the perspective of the visually impaired person, or on global rehabilitation goals and outcome measures (e.g. [7-18]). Also in the Netherlands, most studies focus on specific domains of rehabilitation such as mobility, adjustment or reading/fine work, or on other important outcome measures, such as vision-related quality of life [19-22].

However, MRCs lack an instrument which examines and prioritises specific rehabilitation needs in a systematic way. Such an instrument would also enable more effective evaluation of rehabilitation outcome. Therefore, we developed an adapted and extended Dutch version of the Activity Inventory (AI) by Massof et al. [23,24], the so-called Dutch ICF Activity Inventory (D-AI). This instrument not only prioritises rehabilitation needs at the goal level, but also assesses the difficulty of specific tasks that belong (from the patient's perspective) to relevant goals.

MRCs in the Netherlands are planning to make their intake procedure more structured and objective and showed interest in using the D-AI for this purpose. Because implementation of a new intake method needs to be performed accurately and gradually, an important step in this process is a feasibility study; however, this type of study shows considerable variety in both purpose and methods [25-30].

To investigate rehabilitation needs, this limited pilot study (as part of a longer-term investigation): i) tests the feasibility of the D-AI using a computer-assisted telephone interview (CATI), ii) determines whether the most relevant topics are included, and (iii) tests whether all questions and answer categories are clear and satisfactory. For this purpose, patients and assessors were asked about their perceptions of and experiences with the D-AI using a CATI.

## Methods

### Recruitment of the study population

To assess feasibility of the questionnaire in visually impaired elderly, eligible participants had to be at least 50 years old, speak adequate Dutch, and have sufficient cognitive ability to participate. Persons with low vision from any cause were allowed to participate.

Patients were recruited after enrolment at a specific location of an MRC. All patients met the study criteria as defined in the evidence-based guidelines on the referral of visually impaired persons to low-vision services in the Netherlands [3]. Following this Dutch guideline implies that the inclusion of patients was not limited to only those who strictly meet the formal criteria for low vision as defined by the WHO. For example, also patients with substantial visual field loss, problems with low or high light levels, or severe problems with reading were allowed to participate.

A random sample of 32 patients was taken from patients who had enroled between September and November 2007. Data collection took place before the start of rehabilitation (i.e. between November 2007 and January 2008).

The study protocol was approved by the Medical Ethics Committee of the VU University Medical Center Amsterdam and was consistent with the principles of the Declaration of Helsinki. All patients provided written informed consent.

The D-AI was administered by four different assessors, including the first author who trained the other three assessors. These three assessors were employees of the MRC where the pilot study took place and were usually involved in the intake process. After the first interview was conducted by the first author, the other three assessors conducted nine, seven and three interviews, respectively.

### Structure and routing of the D-AI

The D-AI consists of many possible rehabilitation goals. Each goal belongs to one of the 'Activity and Participation' chapters of the International Classification of Functioning, Disability and Health (ICF) [31]. Additionally, each goal consists of different tasks that all serve the same goal. For each goal the importance is rated on a scale of 0 (not important) to 3 (very important). Only if the goal is at least 'a little important' (goal importance score: 1) will the difficulty of this goal, caused by the visual impairment, be rated on a scale of 0 (not difficult) to 4 (impossible). Only tasks of goals that are at least 'a little important' (goal importance score: 1) and 'a little difficult' (goal difficulty score: 1) will be fully assessed at the task level. Additionally, these goals are labelled with a priority score higher than zero (priority score = goal importance score * goal difficulty score).

Five hobby-related goals (e.g. creative activities) consist of sub-goals. These sub-goals will only be assessed if the overall goal has a priority score higher than zero. In order to develop the first draft of the D-AI, a literature study was made, and patient records and focus group discussions were employed. This process and the content of the D-AI are described in detail elsewhere by Bruijning et al. [32].

### Data collection

The D-AI and its routing (see above) were programmed using Blaise Enterprise 4.7 (Heerlen, the Netherlands), so that it could easily be assessed using a CATI. Depending on the patient's response, the computer automatically displays the following question. Immediately after assessing the D-AI, participants were asked to complete questions about the D-AI and its assessment, with the aim to improve the feasibility of the questionnaire. These questions were: What do you think about the assessment time? How did you feel about completing the questionnaire? What other activities or rehabilitation needs do you suggest should be included? How do you feel about completing the questionnaire by telephone? Do you have any recommendations to improve the questionnaire?

The three assessors who were usually involved in the intake process were also requested to fill out an evaluation form about the D-AI. Additionally, the assessors reported each item which they thought was (possibly) misunderstood. Finally, additional recommendations about the content and wording of the D-AI were requested from one expert (an occupational therapist with a PhD in rehabilitation of visually impaired adults).

Patient characteristics, such as age, visual acuity and eye condition, were collected from the patient's medical files.

### Analysis

For all goals, priority scores were calculated, as well as the mean, median and maximum scores of goal importance, goal difficulty and priority scores. Data were analysed using SPSS 15.0. If possible, similar answers on the evaluation forms were combined. Finally, based on the results of this pilot study, each item was discussed and consensus between the first and second authors was reached with the aim to develop an improved version of the D-AI.

## Results

### Study population

Of the 32 patients randomly selected to be invited to join this pilot study, 20 (62.5%) agreed to participate. Table 1 shows the reasons for not participating. All patients who agreed to participate completed the study. Table 2 presents the characteristics of the patient group.

**Table 1:** Reasons for non participation in the study (n = 12).

	Number of patients (%)
Would take too much effort*	5 (41.7)
Seemed to be cognitively unable to participate	3 (25)
Died between the enrolment and the invitation.	1 (8.3)
Patient felt the regular intake process was too slow and only wanted to participate after rehabilitation	1 (8.3)
Patient could not be invited because personal, ophthalmic and/or other medical information was lacking	1 (8.3)
Reason was unclear (the patient's son made the decision)	1 (8.3)

MRC: Multidisciplinary Rehabilitation Centre

* Two (16.7%) of these patients were participating in another study at this MRC

**Table 2:** Characteristics of the patient group (n = 20).

Characteristics (Specification)	Number of participants (%)	
**Age in years**		
Mean (range)	70.75 (51-89)^$^	

**Sex**		
Male	8 (40)	

**Eye condition (as reported in patient file)**		
Macular degeneration	8 (40)	
Cataract	5 (25)	
Glaucoma	2 (10)	
Refractive error (severe)	2 (10)	
Neurological problem	2 (10)	
Arterial problem	2 (10)	
Cornea diseases	1 (5)	
Retinitis pigmentosa	1 (5)	
Amblyopia	1 (5)	
Unknown	1 (5)	
More than one eye condition	5 (25)	

**Visual Ability**		
**Visual Acuity **(as reported in patient file*)	**better eye**	**worse eye**
VA in Snellen: ≥ 0.5	5 (25)	0 (0)
VA in Snellen: ≥ 0.3 - 0.5	5 (25)	1 (5)
VA in Snellen: ≥ 0.1 - 0.25	6 (30)	6 (30)
VA in Snellen: ≥ 0 - 0.09	4 (20)	12 (60)
**Substantial visual field loss**	6 (12)	7 (35)
**Worse eye interferes with better eye**^**#**^	3 (15)	
**Difficulty with low or high light levels**	3 (15)	
**Rapid fatigue of eyes**	2 (10)	

* If more than one vision test was applied to the patient, the most recent data were used. Data of four patients were obtained by the ophthalmologist (measured with own correction). Data of one patient was obtained by another MRC (measured with the best possible correction). Data of 15 patients were obtained by the low vision specialist of the MRC (measured without correction; for 9 patients a better correction was not possible).

^# ^When looking with both eyes

$ Mean (range) in stead of 'Number of participants (%)'

### Evaluation of the D-AI

The mean time to complete the telephone interview for the D-AI was 88.8 (± 41.0; range 35-180) min.

The mean number of main goals and sub-goals fully assessed at the task level (with a priority score > 0) was 26.5 (± 15.3). If sub-goals are excluded, this number decreases to 22.7 (± 13.8). For one participant only eight main/sub-goals were fully assessed. The highest number of main goals and sub-goals that were fully assessed was 62.

Further analysis of the assessment of the D-AI showed that for 21 main goals and for 38 sub-goals the mean priority score was less than one. There were four main goals (and eight sub-goals) for which none of the participants had a priority score of at least one. To give an impression of the data, Table 3 presents the most important characteristics of the goals, e.g. the mean priority score of each goal. Characteristics of the sub-goals are presented in Table 4. Some sub-goals were given similar answers by the same participants (e.g. 'painting' and 'drawing').

**Table 3:** D-AI characteristics of the goals.

A&P of ICF	**Goal of D-AI**_**pilot**_	**No. of items D-AI**_**pilot**_	No. of items in D-AI	Mean GI	Mean GD	Mean PR	Median PR	Max PR	Patients (n) with PR > 1	Item order D-AI
**1**	Reading	13	19	2.6	2.6	7.3	8.5	12	18	1
	Writing	6	6	2.7	2.0	5.5	4	12	17	2
	Watching TV	10	18	2.6	2.3	5.9	6	9	19	3

**2**	Personal administration	10	11	2.4	2.0	5.8	6	12	14	4
	Follow a schedule	6	6	3.0	1.4	4.1	3	12	12	5

**3**	Using computer at home	23	25	1.3	0.8	2.2	0	12	6	6
	Personal correspondence	8	8	2.6	1.8	5.2	3.5	12	15	7
	Using telephone	7	7	3.0	0.9	2.6	0	12	8	8

**4**	Mobility at home	9	11	3.0	0.4	1.1	0	9	4	9
	Mobility indoors somewhere else	11	11	2.3	1.3	3.0	2	9	12	10
	Walking outdoors	22	28	2.9	1.2	3.6	3	12	11	11
	Driving a vehicle for disabled	24	24	0.4	0.4	0.7	0	9	2	*14*
	Riding a bike	17	17	1.6	0.8	2.3	0	12	7	*12*
	Riding a motorised bike/scooter	17	17	0.6	0.2	0.5	0	9	1	*13*
	Driving a car	24	25	0.8	0.1	0.3	0	3	2	15
	Using public transportation	16	18	1.8	1.3	3.8	0	12	9	16

**5**	Dressing	13	13	3.0	0.7	2.1	0	12	8	17
	Personal hygiene	20	16	2.8	1.2	3.4	3	12	13	18
	Using a public toilet	8	8	1.8	1.1	3.2	0	12	9	19
	Personal heath care	24	24	2.9	0.5	1.4	0	12	4	20
	Eating and drinking	11	14	2.7	0.4	1.2	0	9	5	21

**6**	Household tasks	16	16	2.5	1.2	3.5	3	12	12	22
	Doing laundry	8	9	2.3	0.4	1.2	0	12	3	23
	Doing chores at home	11	11	1.3	1.0	2.6	0	12	7	24
	Mending clothes	6	6	1.3	0.9	2.6	0	12	7	25
	Withdraw or dealing with money	9	10	2.6	1.2	3.5	0	12	8	26
	Daily shopping	27	28	2.7	1.2	3.4	1	12	10	27
	Daily meal preparation	8	28	2.0	0.7	1.8	0	12	5	28
	Guide dog care	9	10	0.3	0.0	0.0	0	0	0	29
	Pet care	12	12	0.6	0.0	0.0	0	0	0	30
	Shopping	4	5	2.2	1.3	3.6	2.5	12	11	31
	Health care for an adult	21	21	1.4	0.7	2.0	0	12	5	32
	Child care	15	15	2.1	0.3	0.7	0	9	2	33

**7**	Recognition and communication	16	16	3.0	0.4	1.1	0	9	5	34
	Interaction with partner	8	8	2.0	0.2	0.5	0	6	2	35
	Interaction with family	8	7	2.6	0.2	0.5	0	9	1	36
	Interaction with relatives and friends	4	4	2.8	0.1	0.3	0	6	1	37
	Interaction with colleagues	15	15	0.9	0.1	0.2	0	3	1	38
	Interaction with strangers	6	6	2.1	0.2	0.4	0	3	4	39

**8**	Manage finance	13	13	2.2	0.9	2.3	0	12	6	40
	Make ends meet	4	4	2.2	0.5	1.1	0	9	4	41
	Regulatory and information	8	9	2.8	1.6	4.7	5	12	12	42
	Education	12	12	0.8	0.5	1.1	0	9	4	43
	Apply for a job	5	5	0.5	0.0	0.0	0	0	0	44
	Accessibility at work	9	9	0.8	0.2	0.5	0	3	3	45
	Working activities	12	14	0.9	0.1	0.3	0	6	1	46
	Using computer at work	22	4	0.5	0.0	0.0	0	0	0	47
	Attend meetings	12	11	0.5	0.2	0.3	0	3	2	48

**9**	Follow the news	16	16	2.5	0.8	2.2	0	9	8	49
	Intellectual activities	11	11	0.6	0.3	0.8	0	9	3	50
	Having visitors	17	16	2.1	0.8	2.0	0	9	7	51
	Social events	24	30	1.8	1.1	2.7	0	12	8	52
	Dining out	19	19	1.2	0.5	1.1	0	12	4	53
	* Social activities and trips	3		1.8	0.9	2.0	0	12	6	
	Going on holiday	14	14	1.9	1.0	2.8	0	12	6	*54*
	Gardening	12	12	1.8	0.7	1.6	0	12	8	*55*
	Making music	4	4	0.2	0.4	0.7	0	9	2	*56*
	Perform in public	4	4	0.2	0.1	0.2	0	3	1	*57*
	* Watching TV or film (recreational)	7		2.9	2.4	6.8	7.5	12	18	
	* Using specific ICT tools	6		1.1	0.5	1.5	0	9	4	
	Attend cultural events	5	5	0.9	0.4	0.9	0	9	4	*58*
	Playing games	2	2	1.2	0.5	0.9	0	6	5	*59*
	Creative activities	1	1	0.9	0.7	1.9	0	12	5	*60*
	Hobbies and crafts	6	6	0.5	0.4	1.1	0	12	2	*61*
	Play sports	8	8	2.3	0.5	1.2	0	6	7	*62*

**10**^**#**^	Feeling fit	9	11	3.0	1.1	3.3	3	9	15	*63*
	Handle feelings	11	11	2.9	1.6	4.3	3.5	9	15	*64*
	Acceptance	15	18	2.9	1.5	4.2	3	9	14	*65*

A&P of ICF: Activity and Participation domain of the International Classification of Functioning, Disability and Health (ICF):

1 Learning and applying knowledge; 2 General tasks and demands; 3 Communication; 4 Mobility; 5 Self-care; 6 Domestic life; 7 Interpersonal interactions and relationships; 8 Major life areas; 9 Community, social, and civic life; 10# Mental Health (emotional) aspects.

D-AI_pilot_: the first draft of the D-AI (Dutch ICF Activity Inventory) which was used for the pilot study.

D-AI: the new version of the D-AI (Dutch ICF Activity Inventory) after the adaptations were made.

# nd-mh: not definable-mental health as stated by Cieza. 2005 [34]. No Activity and Participation domain of the ICF.

GI: goal-importance question score; GD: goal-difficulty question score; PR: priority score.

Column 'Item order D-AI': *Italic *numbers represent a change in the order compared to the D-AI_pilot_.

* means that, after the pilot study, this goal has been deleted or merged with another goal. Some underlying activities were merged with other goals.

**Table 4:** D-AI characteristics of the sub-goals.

**Goal of D-AI**_**pilot**_	**No. of items D-AI**_**pilot**_	Mean PR	Max PR	Patients (n) with PR > 1	Main goals of D-AI sub-goals of D-AI	No. of items D-AI
**Attend cultural events**	5	0.9	9	4	**Attend cultural events**	5
going to a theatre play	3	0.8	9	4	going to the theatre (e.g. plays, cabaret, dancing, music)	3
going to a dance show	3	0.1	1	1	*	
going to a theatre	3	0.1	1	1	*	
attend a concert	3	0.8	9	4	*	
going to the cinema	3	0.2	3	2	going to the cinema	3
attend a museum	1	1.1	9	3	attend a museum	1
attend sporting events	-	0.7	9	2	attend sporting events	2

**Playing games**	2	0.9	6	5	**Playing games**	2
play playing cards games (alone)	2	1.1	9	4	play playing cards games	3
play playing cards games (together)	2	1.0	6	4	*	
play board games	1	0.5	4	3	play board games or puzzles	1
make a jigsaw/puzzle	1	0.6	12	1	*	
playing word games	0	0.8	9	2	playing bingo or word games (e.g. crossword puzzles)	2
playing bingo	1	0.3	6	1	*	
playing computer games	0	0.3	6	1	playing computer games	2

**Creative activities**	1	1.9	12	5	**Creative activities**	1
carving	3	0.0	0	0	moulding or carving	3
moulding	3	0.0	0	0	*	
painting	6	1.0	12	2	painting or drawing	6
drawing	6	1.0	12	2	*	
model building	5	0.6	12	1	model building or crafts	5
crafts	5	1.6	12	5	*	
photography	12	1.2	12	2	photography or using photo software	12
using photo software	12	1.1	12	2	*	
collect things	3	0.3	6	1	collect things	3
needlework	10	1.0	9	3	needlework	10

**Hobbies and crafts**	6	1.1	12	2	**Hobbies and crafts**	6
woodworking	11	0.2	3	1	woodworking	11
metalwork	7	0.6	12	1	metalwork	7
electrical work	5	0.6	12	1	electrical work	5
general household maintenance tasks	13	0.6	12	1	general household maintenance tasks	13

**Play sports**	8	1.2	6	7	**Play sports or physical exercises**	8
walking (going for a walk)	0	1.4	9	5	(Nordic) walking or jogging/running	2
Nordic walking	0	0.0	0	0	*	
jogging or running	0	0.0	0	0	*	
sport cycling	0	0.6	12	1	sport cycling	2
swimming	0	0.7	12	2	swimming	4
riding a boat	0	0.6	12	1	*	
fishing	0	0.6	12	1	fishing	1
skiing	0	0.2	4	1	*	
yoga	0	0.0	0	0	*	
aerobics	0	0.0	0	0	fitness, aerobics or strength training	2
fitness	2	0.0	0	0	*	
strength training	2	0.0	0	0	*	
ball sports	4	0.4	8	1	ball or racket sports (e.g. soccer, golf, tennis, bowling, track ball)	4
golf	4	0.6	12	1	*	
bowling	4	0.7	12	2	*	
play croquet	4	0.6	12	1	*	
racket sports	4	0.6	12	1	*	
playing team sports	2	0.2	4	1	playing team sports	2
dancing	3	0.5	6	2	dancing (e.g. folk or ballroom dancing)	3

D-AI_pilot_: the first draft of the D-AI (Dutch ICF Activity Inventory), which was used for the pilot study.

D-AI: the new version of the D-AI (Dutch ICF Activity Inventory), after the adaptations were made.

GI: goal-importance question; GD: goal-difficulty question; PR: priority.

Column 'Goal of D-AI_pilot_': **Bold **phrases indicate the main goals that contain sub-goals.

* means that this goal has been or merged with another goal. Some underlying activities were merged with other goals.

### Feedback from assessors and patients

The three assessors indicated that the D-AI is a more objective way to investigate rehabilitation needs and that it is a practical instrument to systematically assess the most important and difficult goals and tasks for patients. No topics are overlooked and the quality of the intake is less dependent on the characteristics of the intake assessor. Patients and assessors thought there was no need to include or delete any specific item. However, they felt that some questions needed rephrasing to improve the clarity. For example, some assessors reported that in many cases the goal 'social activities and trips' was initially interpreted as 'social events' or as 'going on holiday'. Additionally, 'watching TV or film (recreational)' appeared to be too similar to 'watching TV'. Also, the goal 'using specific ICT tools' was reported to be misunderstood. One assessor suggested that is would be useful to establish what percentage of the interview was completed during the CATI.

All three assessors agreed that the D-AI interview took too much time, which was 'uncomfortable' and 'unpractical'. Furthermore, they noted that some questions showed up two or more times, which could be annoying for the participants. Moreover, they suggested to include 'not applicable' as an answer category for the question on goal importance. Additionally, one assessor reported that the systematic character and multiple-choice options of the questionnaire made spontaneous conversation difficult and thought that the patients may not like this.

A summary of the patient evaluation is presented in Table 5.

**Table 5:** Evaluation by the patients (n = 20).

Item	Opinion	Number of participants (%)	Statement
**General opinion about the questionnaire assessment**	+	10 (50)	*- Nice; Fun; Interesting; Useful.*
	±	9 (45)	*- Neutral; Not unpleasant; Did not care.*
	-	1 (5)	*- Ineffective.*

**Opinion about administration time**	-	12 (60)	*- Too long; Lack of concentration; Physical pain (e.g. due to holding telephone); Tiring (need break).*
	+	6 (30)	*- Fine; Good.*
	±	2 (10)	*- Nothing special.*

**What did you think of the administration by telephone?**	+	14 (70)	*- Fine; Perfect; Pleasant.*
	±	4 (20)	*- No comment; Not unpleasant.*
	-	2 (10)	*- Face-to-face would be better; Physical pain (e.g. due to holding telephone); Tiring.*

**Any activities or rehabilitation needs that were not included?**	+	17 (85)	*- No additions; Everything was included.*
	*	3 (15)	*- No further comments.*

**Any other recommendations?**	-	11 (55)	*- Some items were administered twice (e.g. reading).*
	-	3 (15)	*- Some goals are important, but at the same time 'not applicable'.*
	*	6 (30)	*- No further comments.*

**Unclear questions reported by patient**	+	11 (55)	*- All questions were clear.*
	*	9 (45)	*- No further comments.*

+: positive opinion

±: neutral opinion

-: negative opinion

*: no further comments

### Adaptations to the D-AI

After analysing the results, some minor changes were made to the order of the items. Furthermore, three main goals, 'social activities and trips', 'watching TV or film (recreational)' and 'using specific ICT tools' were deleted. Some tasks that belonged to these goals were moved to other goals. For example, the item 'watching news programs' initially belonging to the goal 'watching TV or film (recreational)' was moved to the goal 'watching TV'. Finally, some tasks were added, deleted or rephrased. A summary of the results is given in Tables 3 and 4.

Furthermore, the answer category 'not applicable' has been added to the question on goal importance. Also, an explanation of this answer category is now included in the introduction of the D-AI, as follows: *'Not applicable' means that you usually do not perform this subject, either because of physical conditions other than your eye condition, or because you are satisfied with the help you get concerning this subject.*

**Figure 1:**
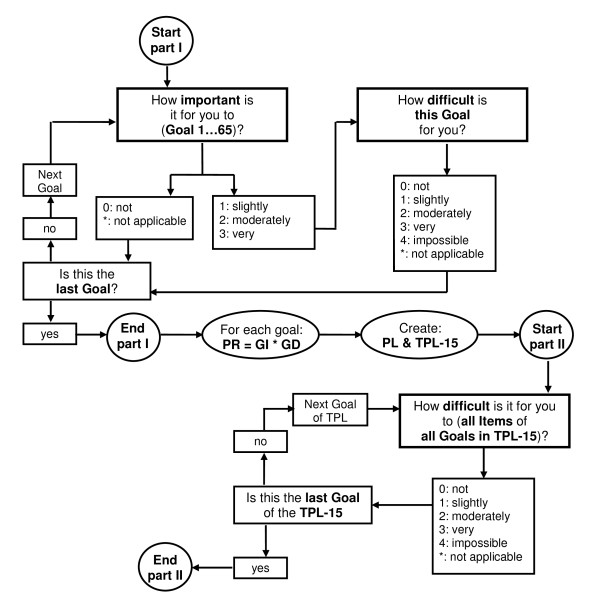
**Routing of the new version of the D-AI**. PR: goal priority score. GI: goal importance score. GD: goal difficulty score. PL: priority list in which all goals are ranked from the highest to the lowest priority score. TPL-15: top priority list which contains all the goals with the same or a higher priority score as the fifteenth goal of the priority list.

Another important change concerns the structure of the D-AI. The new version consists of two parts. First, for all goals the 'importance' question and - if the goal is at least 'a little important' (importance score: 1) - the 'difficulty' question will be assessed. Priority scores of all goals are then determined and a priority list (PL) composed, in which all goals are ranked from the highest to the lowest priority score. The second part of the questionnaire consists of assessing all tasks that belong to the goals that have the same or a higher priority score as the fifteenth goal of the list (TPL-15). This means that not all goals with a priority score of at least one will be fully assessed. However, this is possible if the goal with the lowest priority score has the same score as the fifteenth goal. The mean number of goals (including sub-goals) per person that would have been administered following the new structure (TPL-15) is 18.75 ± 6.3 (range 8-34). Figure 1 shows this new structure and routing.

## Discussion

### Feasibility of the D-AI

The results of this study support the feasibility of the extended and adapted D-AI for use with visually impaired older adults, assessed by a CATI. In general, participants and assessors were positive about the content of the D-AI and both groups reported that the topics in the D-AI covered all relevant rehabilitation needs. As was confirmed by the assessors, the D-AI had some additional important positive qualities: they felt that the D-AI is a more objective way to assess rehabilitation needs, it makes the intake less dependent on the individual qualities of the intake assessor, and prevents important topics being overlooked by the patient and/or the intake assessor.

In this population of visually impaired older adults, a CATI was considered to be an appropriate way to assess the D-AI. Moreover, direct data entry limits the non-response rate to questionnaire items, because of the enforcement of answers. Additionally, it facilitates compiling large databases for the items, which will increase possibilities for future analyses [33]. Most participants were positive about the assessment by telephone, although one patient preferred a face-to face-interview. In the future, different assessment methods can be applied, depending on the patient's preference. Although most participants were positive about the interview, many of them (as well as all assessors) reported that the D-AI took too long to administer. Two participants even needed a break because they had physical pain (e.g. due to holding the telephone) or could not sustain concentration. The new version of the D-AI, in which only those goals with top priority scores will be fully assessed, is expected to increase feasibility as it will decrease the administration time.

The threshold of the TPL-15 was chosen after comparing the assessment time with the number of assessed goals. We expect that this cut-off will decrease the administration time by 15 min. More important, we expect it to lower the outliers with a long assessment time and, simultaneously, to maintain a suitable amount of detailed information. However, because the exact threshold of 15 was arbitrary, more studies are needed to establish whether assessing only those tasks that belong to the goals that have the same or higher priority score as the fifteenth goal of the list, is sufficient to investigate the rehabilitation needs of the visually impaired elderly.

### Content and formulation of the D-AI

All assessors and eleven participants reported that some (similar) task questions were asked two or more times (e.g. reading similar items). Examples are 'reading a TV guide' or 'reading a medicine label'; however, these comparable items belonged to different goals (e.g. 'watching TV' and 'personal healthcare'). Not all goals will be assessed and, because the difficulty level of these similar items (e.g. reading) might differ in another context, these double items have not yet been excluded. In an upcoming validation study with a much larger population, we will examine these similar items with the aim to include only the most distinct items.

Another important change was adding an extra answer category to the goal-importance question. Patients complained that the answer categories did not always reflect how they felt. For instance, the goal 'household tasks' was often described as 'not applicable' because the participant had a housekeeper. Several participants reported that saying 'not important' did not adequately represent their situation.

As can be seen in Table 3, some goals had very low mean priority scores. However, some of these goals had very high individual priority scores, meaning that this goal was relevant for some individual participants. We consider that the sample size in this study was too small to delete goals based on low mean priority scores alone. None of the participants had a priority score for the following main goals: 'guide dog care', 'pet care', 'apply for a job' and 'using a computer at work'. However, in this pilot study the youngest participant was 51 years old and, because these four goals are probably more relevant for younger persons, they have not been excluded.

### Future implementation

Dutch MRCs are willing to adopt a more structured and objective intake procedure and showed interest in using the D-AI for this purpose. The systematic character of the D-AI prevents topics from being overlooked and indicates which needs have the highest priority from a patient-centred perspective. The results in this study show a wide variety of personal rehabilitation needs. This supports the use of an extensive and systematic instrument that enables each individual to receive their own personal rehabilitation plan. Moreover, ongoing assessment of the D-AI will enhance evaluation of the rehabilitation process. Successful rehabilitation will result in lower priority scores because, after rehabilitation, the goals will be less difficult and/or less important, e.g. due to newly learned alternatives or because of increased acceptance of vision loss. This enables more effective monitoring of the individual patient's progress, as well as evaluation of specific rehabilitation programmes.

Concerning implementation of the D-AI, we suggest that MRCs link all possible rehabilitation goals to specific rehabilitation programmes and assistive devices. After assessment of the first part of the D-AI (at the goal level), the patient and a professional from the MRC will discuss which rehabilitation options will be feasible and where to start. From this viewpoint, not only the 'Activity and Participation' domains but also the total ICF scheme will be discussed. After this, a rehabilitation plan can be formulated together. At the end of the rehabilitation trajectory, this rehabilitation plan will be evaluated to establish whether rehabilitation is successful and, if necessary, to reformulate the plan.

## Conclusions

In conclusion, assessment of the extended and adapted D-AI appears to be a promising way to systematically investigate and evaluate rehabilitation needs immediately after entering an MRC. The results of this feasibility study clearly indicate how to improve the feasibility of the D-AI. The small sample size seemed to be sufficient as the results were relatively homogeneous. The findings in this study support the relevance of further developing the D-AI. The next step in this process is a limited patient file study to compare rehabilitation plans based on the regular intake and on the D-AI. To further improve the D-AI and to be able to report important psychometric properties, a larger validation study will also be conducted.

## List of abbreviations used

D-AI: Dutch ICF Activity Inventory; MRC: Multidisciplinary Rehabilitation Centre; AI: Activity Inventory; CATI: Computer-Assisted Telephone Interview; ICF: International Classification of Functioning, Disability and Health; PL: Priority List; TPL-15: Top Priority List

## Competing interests

The authors declare that they do not have competing interests. Moreover, the sponsor of the study had no role in the design and conduct of the study, the data collection, data analysis, data interpretation, or writing of the report.

## Authors' contributions

JB participated in the design, the acquisition, analysis and interpretation of data, and drafted the manuscript. RvN and GvR participated in the design of the study and were involved in drafting the manuscript or critically revising it for important intellectual content. All authors have read and approved the final manuscript.

## Pre-publication history

The pre-publication history for this paper can be accessed here:

http://www.biomedcentral.com/1472-6963/10/318/prepub

## References

[B1] Congdon N O'Colmain B Klaver CC Klein R Munoz B Friedman DS Kempen J Taylor RH Mitchell P Causes and prevalence of visual impairment among adults in the United States Arch Ophthalmol 2004 122 477 485 10.1001/archopht.122.4.477 15078664

[B2] Limburg H Epidemiologie van visuele beperkingen en een demografische verkenning 2007 [Epidemiology of visual disabilities and a demographic investigation]; Report commissioned by the Netherlands organization for health research and development (ZonMw) and the InSight Society

[B3] de Boer MR Langelaan M Jansonius NM Van Rens GHMB Evidence-based guidelines on the referral of visually impaired persons to low vision services Eur J Ophthalmol 2005 15 400 406 15945011 10.1177/112067210501500314

[B4] Leach E Cornwell P Fleming J Haines T Patient centered goal-setting in a subacute rehabilitation setting Disabil Rehabil 2009 1 14 10.1080/09638280903036605 19562579

[B5] Pollock N Client-centered assessment Am J Occup Ther 1993 47 298 301 8322870 10.5014/ajot.47.4.298

[B6] Siegert RJ McPherson KM Taylor WJ Toward a cognitive-affective model of goal-setting in rehabilitation: is self-regulation theory a key step? Disabil Rehabil 2004 26 1175 1183 10.1080/09638280410001724834 15371017

[B7] Boerner K Cimarolli VR Optimizing rehabilitation for adults with visual impairment: attention to life goals and their links to well-being Clin Rehabil 2005 19 790 798 10.1191/0269215505cr893oa 16250199

[B8] Wolffsohn JS Cochrane AL Design of the low vision quality-of-life questionnaire (LVQOL) and measuring the outcome of low-vision rehabilitation Am J Ophthalmol 2000 130 793 802 10.1016/S0002-9394(00)00610-3 11124300

[B9] Mangione CM Lee PP Pitts J Gutierrez P Berry S Hays RD Psychometric properties of the National Eye Institute Visual Function Questionnaire (NEI-VFQ). NEI-VFQ Field Test Investigators Arch Ophthalmol 1998 116 1496 1504 9823352 10.1001/archopht.116.11.1496

[B10] Stelmack J Quality of life of low-vision patients and outcomes of low-vision rehabilitation Optom Vis Sci 2001 78 335 342 10.1097/00006324-200105000-00017 11384011

[B11] Stelmack JA Stelmack TR Massof RW Measuring low-vision rehabilitation outcomes with the NEI VFQ-25 Invest Ophthalmol Vis Sci 2002 43 2859 2868 12202503

[B12] Rees G Saw CL Lamoureux EL Keeffe JE Self-management programs for adults with low vision: needs and challenges Patient Educ Couns 2007 69 39 46 10.1016/j.pec.2007.06.016 17686604

[B13] Stelmack JA Massof RW Using the VA LV VFQ-48 and LV VFQ-20 in low vision rehabilitation Optom Vis Sci 2007 84 705 709 10.1097/OPX.0b013e3181339f1a 17700334

[B14] O'Connor PM Lamoureux EL Keeffe JE Predicting the need for low vision rehabilitation services Br J Ophthalmol 2008 92 252 255 10.1136/bjo.2007.125955 18227205

[B15] Lamoureux EL Pallant JF Pesudovs K Tennant A Rees G O'Connor PM Keeffe JE Assessing participation in daily living and the effectiveness of rehabiliation in age related macular degeneration patients using the impact of vision impairment scale Ophthalmic Epidemiol 2008 15 105 113 10.1080/09286580701840354 18432494

[B16] Lorenzana L Lankaranian D Dugar J Mayer J Palejwala N Kulkarni K Warrian K Boghara Z Richman J Wizov S Spaeth G Almodin J A new method of assessing ability to perform activities of daily living: design, methods and baseline data Ophthalmic Epidemiol 2009 16 107 114 10.1080/09286580902738142 19353399

[B17] Gothwal VK Wright TA Lamoureux EL Pesudovs K Visual Activities Questionnaire: assessment of subscale validity for cataract surgery outcomes J Cataract Refract Surg 2009 35 1961 1969 10.1016/j.jcrs.2009.05.058 19878830

[B18] Marella M Gothwal VK Pesudovs K Lamoureux E Validation of the visual disability questionnaire (VDQ) in India Optom Vis Sci 2009 86 E826 E835 10.1097/OPX.0b013e3181ae1b3f 19543138

[B19] van Nispen RMA Knol DL Langelaan M de Boer MR Terwee CB van Rens GHMB Applying multilevel item response theory to vision-related quality of life in Dutch visually impaired elderly Optom Vis Sci 2007 84 710 720 10.1097/OPX.0b013e31813375b8 17700335

[B20] van Nispen R Knol D Neve H van Rens G A multilevel item response theory model was investigated for longitudinal vision-related quality-of-life data J Clin Epidemiol 2010 63 321 330 10.1016/j.jclinepi.2009.06.012 19766455

[B21] Langelaan M de Boer M van Nispen R Wouters B Moll A van Rens G Change in quality of life after rehabilitation: prognostic factors for visually impaired adults Int J Rehabil Res 2009 32 12 19 10.1097/MRR.0b013e3283063503 19057390

[B22] de Boer MR Moll AC de Vet HCW Terwee CB Volker-Dieben HJM van Rens GHMB Psychometric properties of vision-related quality of life questionnaires: a systematic review Ophthalmic Physiol Opt 2004 24 257 273 10.1111/j.1475-1313.2004.00187.x 15228503

[B23] Massof RW Hsu CT Baker FH Barnett GD Park WL Deremeik JT Rainey C Epstein C Visual disability variables. I: the importance and difficulty of activity goals for a sample of low-vision patients Arch Phys Med Rehabil 2005 86 946 953 10.1016/j.apmr.2004.09.016 15895341

[B24] Massof RW Hsu CT Baker FH Barnett GD Park WL Deremeik JT Rainey C Epstein C Visual disability variables. II: The difficulty of tasks for a sample of low-vision patients Arch Phys Med Rehabil 2005 86 954 967 10.1016/j.apmr.2004.09.017 15895342

[B25] Watson PD Denny SJ Adair V Ameratunga SN Clark TC Crengle SM Dixon RS Fa'asisila M Merry SN Robinson EM Sporte AA Adolescents' perceptions of a health survey using multimedia computer-assisted self-administered interview Aust N Z J Public Health 2001 25 520 524 10.1111/j.1467-842X.2001.tb00316.x 11824987

[B26] Stuck AE Elkuch P Dapp U Anders J Iliffe S Swift CG Feasibility and yield of a self-administered questionnaire for health risk appraisal in older people in three European countries Age Ageing 2002 31 463 467 10.1093/ageing/31.6.463 12446293

[B27] Page D Weaver F Wilkie D Simuni T A computerized survey of pain in Parkinson's disease patients: A pilot feasibility study Parkinsonism Relat Disord 2010 16 139 141 10.1016/j.parkreldis.2009.07.001 19640770

[B28] White MJ Stark JR Luckmann R Rosal MC Clemow L Costanza ME Implementing a computer-assisted telephone interview (CATI) system to increase colorectal cancer screening: a process evaluation Patient Educ Couns 2006 61 419 428 10.1016/j.pec.2005.05.008 15993558

[B29] Rogausch A Sigle J Seibert A Thuring S Kochen MM Himmel W Feasibility and acceptance of electronic quality of life assessment in general practice: an implementation study Health Qual Life Outcomes 2009 7 51 10.1186/1477-7525-7-51 19493355 PMC2698929

[B30] Chinman M Hassell J Magnabosco J Nowlin-Finch N Marusak S Young AS The feasibility of computerized patient self-assessment at mental health clinics Adm Policy Ment Health 2007 34 401 409 10.1007/s10488-007-0120-4 17453332

[B31] World Health Organisation International classification of functioning, disability and health 2001 Geneve

[B32] Bruijning JE van Nispen RMA van Rens GHMB A Dutch ICF version of the Activity Inventory: results from focus groups with visually impaired persons and experts Ophthalmic Epidemiol 2010 17 366 377 10.3109/09286586.2010.528133 21090911

[B33] Hahn EA Cella D Bode RK Gershon R Lai JS Item banks and their potential applications to health status assessment in diverse populations Med Care 2006 44 S189 S197 10.1097/01.mlr.0000245145.21869.5b 17060827

[B34] Cieza A Geyh S Chatterji S Kostanjsek N Ustun B Stucki G ICF linking rules: an update based on lessons learned J Rehabil Med 2005 37 212 218 10.1080/16501970510040263 16024476

